# Regulated Surface Morphology of Polyaniline/Polylactic Acid Composite Nanofibers via Various Inorganic Acids Doping for Enhancing Biocompatibility in Tissue Engineering

**DOI:** 10.1186/s11671-020-03457-z

**Published:** 2021-01-06

**Authors:** Rongtao Liu, Shiyang Zhang, Chen Zhao, Dong Yang, Tingting Cui, Yidong Liu, Yonggang Min

**Affiliations:** 1 School of Materials and Energy, Guangdong University of Technology (GDUT), Guangzhou, 510006 China; 2 Dongguan South China Design Innovation Institute, Dongguan, 523808 Guangdong China

**Keywords:** Regulated surface morphology, Enhanced biocompatibility, PANI/PLA composite nanofibrous scaffolds, Inorganic acid dopant, Tissue engineering

## Abstract

Conductive and degradable nanofibrous scaffolds have great potential in promoting cell growth, proliferation, and differentiation under an external electric field. Although the issue of inferior electrical conductivity in body fluids still exists, polyaniline (PANI)-based degradable nanofibers can promote cell adhesion, growth, and proliferation. To investigate whether the effect is caused by the PANI morphology, we selected three inorganic acids as dopants in the process of PANI in situ oxidative polymerization: hydrochloric acid, sulfuric acid, and perchloric acid. The obtained polyaniline/polylactic acid (PANI/PLA) composite nanofibers were characterized via SEM, FTIR, and XPS analysis, and we confirmed that the PLA nanofibers were successfully coated by PANI without any change to the porous structure of the PLA nanofibers. The in vitro mechanical properties and degradability indicated that the oxidation of acid dopants should be considered and that it was likely to have a higher oxidation degradation effect on PLA nanofibers. The contact angle test demonstrated that PANI/PLA composite nanofibers with different surface morphologies have good wettability, implying that they meet the requirements of bone tissue engineering scaffolds. The surface roughness and cell viability demonstrated that different PANI morphologies on the surface can promote cell proliferation. The higher the surface roughness of the PANI, the better the biocompatibility. Consequently, the regulated surface morphology of PANI/PLA composite nanofibers via different acids doping has positive effect on biocompatibility in tissue engineering.

## Introduction

Extracellular matrix (ECM) is a type of macromolecular network secreted by cells into the extracellular stroma. It presents the foundation of cells, tissues, and organ, accompanied with organs, and is characterized by a complex grid structure [1, 2]. Moreover, it provides a suitable site for the survival and activity of cells, determining their shape, controlling their differentiation, participating in their migration and metabolism, and ultimately affecting their survival, growth, and death [3, 4]. Electrospinning nanofibers can simulate the action of extracellular matrix to regulate cell behavior due to their high specific surface area, appropriate mechanical properties, and biodegradability. In addition, electrospinning nanofibers can be multifunctional by surface modification on the basis of maintaining its porous structure. Therefore, electrospinning nanofibers have become a promising candidate material in tissue engineering, which is widely applied in drug delivery, orthopedic regeneration, nerve regeneration, and repair [5–10].

Conductive polymers (e.g., polypyrrole [PPy], polythiophene [PTH], and polyaniline [PANI]) have good in vitro and in vivo biocompatibility, which can significantly affect cell adhesion, proliferation, and differentiation as well as tissue regeneration [11–13]. Among these conductive polymers, PANI is considered to be a potential material for tissue engineering and regenerative medicine due to its good processability, excellent conductivity, good redox stability, and biocompatibility [14, 15]. Under electrical stimulation, PANI can regulate cell adhesion, proliferation, migration, and differentiation [16, 17]. In fact, numerous reports have concluded that PANI-based conductive degradable composite nanofibers promote cell behavior under an electric field [18–21]. However, this entails a crucial issue that the conductivity of PANI in physiologic environment (pH = 7.4) will be weakened because of PANI dedoped, which, as previous studies have demonstrated, diminishes its electric activity advantages of promoting cell proliferation and differentiation [22]. While this clearly presents a limitation of PANI-based conductive degradable nanofibers in bone tissue engineering under external electrical stimulation, they can still promote cell proliferation and growth to a significant extent [23, 24]. Here, we speculated that the surface morphology of PANI increases the roughness of the composite nanofibers, which is conducive to cell adhesion, growth, and proliferation.

Polyaniline doped with inorganic acids generally has good electrical conductivity. However, the anions introduced by various inorganic acid dopants will affect the conductivity and structure of polyaniline [25–27]. In this paper, three common inorganic acids, namely hydrochloric acid (HCl, HA), sulfuric acid (H_2_SO_4_, SA), and perchloric acid (HClO_4_, PA), were selected as the dopants in a PANI in situ oxidative polymerization. Then, the mechanical properties, wettability, surface morphology, biocompatibility, and cell adhesion of PANI/polylactic acid (PLA) nanofibers were investigated under different acid dopants. The results indicated that the higher the surface roughness of the PANI, the better the cell proliferation, thus showing better biocompatibility.

## Methods/Experimental

### Chemicals

Aniline (AN) was purchased from Sigma, PLA (*M*w = 60,000) was purchased from Solarbio, dichloromethane (DCM) was purchased from Tianjin Fuyu Fine Chemical Co., Ltd., and N, N-dimethylformamide (DMF) was purchased from Macklin. Meanwhile, ammonium persulfate (APS) was purchased from Aladdin, HCl and H_2_SO_4_ were purchased from Guangzhou Chemical Co., Ltd., and HClO_4_ was purchased from Macklin.

### Preparation of Polyaniline/Polylactic Acid Nanofibers

#### Fabrication of Electrospinning Polylactic Acid Nanofibers

PLA particles with a specific mass were added into a mixed solution of DCM and DMF (volume ratio of 7:3) before being stirred until they dissolved, and a mixed solution of 10% PLA was obtained. The PLA solution was then dispensed into a syringe and connected with a high-voltage power supply. The electrospinning machine (DP-30, Tianjin Yunfan Technology Co., Ltd.) was set with a voltage of 15 kV and at distance of 15 cm. The obtained PLA nanofibers were vacuum-dried overnight at 40 °C.

#### Preparation of Polyaniline/Polylactic Acid Nanofibers Doped with Different Inorganic Acids

**Fig. 1:**
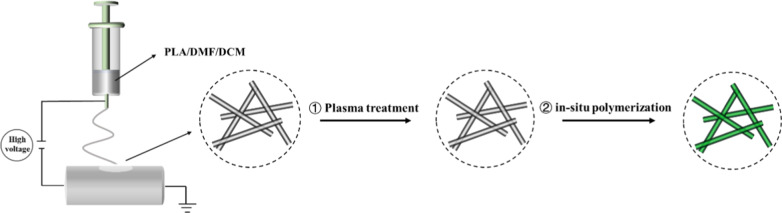
Schematic depicting the preparation process of PLA and PANI/PLA composite nanofibers

The PLA nanofibers were placed in the plasma cleaning machine chamber (PCE-6, MTI Corporation, USA) and discharged for 2 min at 30 W RF power. In this paper, three common inorganic acids, namely HCl, H_2_SO_4_, and HClO_4_, were used as dopants for in situ oxidative polymerization in the preparation of PANI/PLA nanofibers [24], and the corresponding PANI nanofibers were marked as PANI-HA, PANI-SA, and PANI-PA, respectively, while the PANI/PLA nanocomposite nanofibers were labeled as PANI/PLA-HA, PANI/PLA-SA, and PANI/PLA-PA, respectively. The preparation process of PLA and PANI/PLA composite nanofibers is shown in Fig. 1.

PANI/PLA nanocomposite nanofibers were prepared under ice-bath conditions [16, 28]. The APS and AN were added to a 1 M acid solution according to a molar ratio of 1:1. Here, we take the HCl as an example to illustrate the preparation process of PANI/PLA nanofibers. Under ice-bath conditions, the AN (930 mg, 0.01 mol) was added dropwise into APS (2,280 mg, 0.01 mol) and dissolved in 50 mL of 1 M HCl. Immediately, the PLA nanofiber membrane treated by plasma was immersed in the solution and stirred for 2 h at 0 °C. Following the reaction, the PLA nanofiber membrane was cleaned several times with HCl and ethanol to remove the unattached PANI before being dried overnight at 40 °C to obtain PANI/PLA-HA nanofibers, which were placed aside for later use. The PANI/PLA-SA and PANI/PLA-PA composite nanofibers were obtained following a similar approach.

### Characterization

The uniaxial tensile tests for the PLA nanofibers and PANI/PLA composite nanofibers were conducted via a strain–stress test (Shimadzu AGX-PLUS, Japan). Here, the specimen was cut into a dumbbell-like shape, with the tensile speed maintained at a constant 3 mm/min. Young’s modulus was calculated from the 0–15% strain linear region in the stress–strain curve, and the tensile strength and fracture tensile rate of the curve were determined from the fracture of the nanofiber membrane.

The morphology of the nanofiber scaffolds was characterized via field emission scanning electron microscopy (FE-SEM) (Hitachi-SU8220, Japan) to observe the different morphologies of the PANI doped with different inorganic acids. Prior to the SEM observation, the nanofiber samples were sprayed with gold for 60 s to allow for clearer observation of the morphology. Meanwhile, the surface roughness of the PANI/PLA composite nanofibers was measured using atomic force microscopy (AFM, Bruker Dimension Edge). To confirm that PANI was completely loaded on the PLA nanofibers, Fourier transform infrared spectroscopy (FTIR) (Thermo Nicolet iS50) was used to measure the wavelength change of 2000 ~ 500 cm^−1^. X-ray photoelectron spectroscopy (XPS; Thermo ESCALAB 250) and Al-Kα were used as the X-ray emission sources to further determine the surface composition of the PANI/PLA nanofibers, while their wettability was measured in terms of the contact angle of the water droplets at ambient temperature via contact angle analysis (OCA 15 plus, Germany). The degradation of the nanofibers was evaluated using a mass loss method [29, 30]. The nanofiber membranes were cut into 16-mm discs and placed in 20 mL of phosphate buffer saline (PBS) with a pH of 7.4 before the scaffolds were incubated at 37 °C for 7, 14, and 21 days and dried to a constant weight.

### Biocompatibility of PANI/PLA Composite Nanofibrous Scaffolds

#### Biocompatibility

In this paper, the biocompatibility of PANI/PLA composite nanofibrous scaffolds was characterized by human osteosarcoma (HOS) cell activity experiment. HOS cells were purchased from the cell bank of the Chinese Academy of Sciences, Shanghai. The HOS cells were cultured in low-glucose Dulbecco’s modified Eagle medium (DMEM) containing 10% fetal bovine serum, 100 U/mL penicillin, and 100 U/mL streptomycin before being incubated at 37 °C and 5% CO_2_. When the cell growth reached a fusion degree of 90%, the cells were passaged at a ratio of 1:3.

The HOS cells had to be seeded on the PANI/PLA nanofibers prior to the cell proliferation test. Here, the nanofibers were placed into a 96-well plate such that they completely covered the bottom of the plate before being sterilized via UV for 30 min and via a 75% ethanol solution for 30 min. They were then washed with PBS. The nanofibers were then inoculated with a 1 × 10^4^ well density, while a blank group and a control group were simultaneously set up. The cells were then incubated in a cell incubator at 37 °C for one, three, and five days, with the medium refreshed every two days.

Cell viability of the PANI/PLA nanofibers was evaluated using a 3-(4,5-dimethyl-2-thiazolyl)-2,5-diphenyl-2-H-tetrazolium bromide (MTT) assay. Following incubation at one, three, and five days, the medium was removed from the 96-well plate and washed with PBS three times before a 1-mL DMEM containing 10% 5 mg/mL MTT was added. The medium was then incubated at 37 °C for 4 h and then removed before DMSO was added to dissolve the methylprednisolone. The medium was vibrated for 10 min, and then, the absorbance was determined (BioTek Synergy HTX, USA).

#### Fluorescent Immunostaining

The HOS cells were incubated in the PANI/PLA nanofiber incubator for 24 h and were washed with PBS three times. Then, the cells were fixed with 4% paraformaldehyde for 10 min at room temperature. The fixed cells were washed with PBS three times (10 min each time) and 10 μL of 100 nM FITC-labeled peptide was added before the cells were incubated for 30 min at room temperature and then washed with PBS three times (5 min each time). The extracellular actin of the HOS cells was stained, with confocal microscopy (Type A1, Nikon, Japan) used to observe the cell staining at a 20 × magnification.

#### Cell Adhesion

The adhesion of the HOS cells on the PANI/PLA composite nanofibrous scaffolds was observed via SEM. Here, the culture medium was removed after the 24-h PANI/PLA nanofiber HOS cell culture and was then washed with PBS three times before the addition of 4% PFA. The medium was fixed overnight at 4 °C, washed with PBS three times, dehydrated with gradient ethanol solution (30%, 50%, 70%, 85%, 90%, and 100%, respectively; 20 min each time), and then freeze-dried for 24 h. Prior to the SEM observation, the nanofibers were sprayed with platinum for 120 s to allow for better observation.

#### Alkaline Phosphatase Activity (ALP)

ALP is one of the commonly used early osteoblast differentiation markers that depend on alkaline phosphatase enzyme expression. Herein, ALP activity was performed using the ALP Assay Kit (Beyotime Biotechnology, P0321S)**.** The HOS cells were cultured on different PANI/PLA composite scaffolds for the designated 7d. The cells were lysed using 50 μL of Tris–HCl (0.1 M, pH 8) with 0.1% (v/v) triton X-100. The ALP activity is analyzed by quantifying the concentration of *p*-nitrophenol from *p*-nitrophenyl phosphate (PNPP), which is estimated by recording the absorbance at 405 nm. The percentage ALP activity of the cells cultured along the PANI/PLA nanofibers is calculated by comparing the ALP activity of the cells cultured on pristine PLA nanofibers.

### Statistical Analysis

The statistical significance of the results was assessed via one-way analysis of variance (ANOVA) using GraphPad Prism (version 8.02). Here, the differences in mechanical properties, in vitro biodegradability, and cell viability among the different PANI/PLA composite nanofibrous scaffolds were analyzed. The results were considered to be significant when *p* < 0.05 (∗) and very significant when *p* < 0.005 (∗∗).

## Results and Discussion

**Fig. 2:**
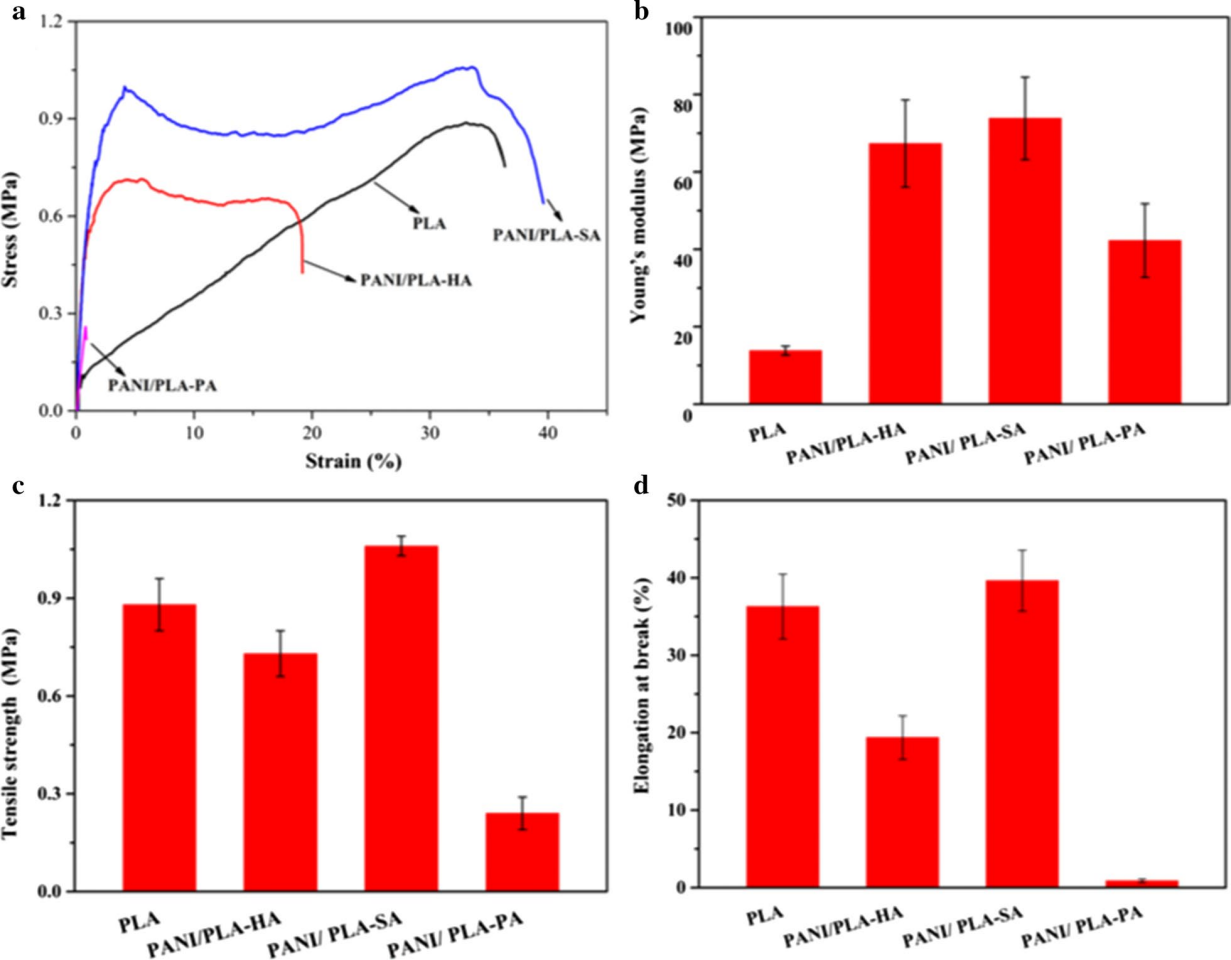
Mechanical properties of PLA nanofibers and PANI/PLA composite nanofibers. **a** Representative tensile stress–strain curves, **b** Young’s modulus, **c** tensile strength at break, **d** elongation at break

The mechanical properties of tissue-engineered scaffolds are important indicators in the evaluation of whether the scaffolds can withstand fluid dynamics. The presence of inorganic acids may affect the physical and chemical properties of the PLA matrix of PANI/PLA composite nanofibers in the in situ chemical oxidation polymerization process of PANI. Therefore, exploring the mechanical properties of PANI/PLA composite nanofibers doped with inorganic acids is necessary. Here, the mechanical properties of PANI/PLA composite nanofibers were evaluated via a tensile test, which is shown in Fig. 2, including stress–strain, Young’s modulus, tensile strength, and elongation at break. As shown in Fig. 2a, the PLA nanofibers exhibited a linear elastic behavior, and the PANI/PLA-HA and PANI/PLA-SA composite nanofibers exhibited clear yield behavior, while the PANI/PLA-PA composite nanofibers broke immediately after elastic deformation. The Young’s modulus (Fig. 2b) of the PANI/PLA composite nanofibers was higher than that of the PLA nanofibers. Compared to PLA, the increase in elastic modulus of PANI/PLA-HA, PANI/PLA-SA, and PANI/PLA-PA was 53.5 ± 9.09, 60.00 ± 9.47, and 28.43 ± 8.34 MPa, respectively. In terms of tensile strength (Fig. 2c) and fracture tensile ratio (Fig. 2d), those of PANI/PLA-HA and PANI/PLA-PA decreased, while those of PANI/PLA-SA slightly increased; the tensile strength and elongation at break of PANI/PLA-PA were the lowest. Compared to the PLA nanofibers, the tensile strength of PANI/PLA-HA and PANI/PLA-PA decreased by 0.15 ± 0.01 and 0.64 ± 0.03 MPa, respectively, while that of PANI/PLA-SA slightly increased by 0.13 ± 0.05 MPa. The elongation at break of PANI/PLA-HA and PANI/PLA-PA decreased by 16.93 ± 1.38% and 35.42 ± 3.94%, respectively, while that of PANI/PLA-SA increased by 3.32 ± 0.13%.

As shown in Fig. 2, the selected inorganic acids can increase the elastic modulus of PLA nanofibers through the connection of the PANI coating. In terms of tensile strength and elongation at break, compared to PLA nanofibers, the mechanical properties of PANI/PLA-HA and PANI/PLA-SA varied to differing degrees, while those of PANI/PLA-PA decreased most clearly, and as soon as the stress was applied during the testing, a fracture occurred in under 5 s. These results may be due to the oxidation of HClO_4_, which resulted in the cleavage of the ester bond in the PLA molecular chain and the oxidative decomposition of the carboxyl group, subsequently leading to inferior mechanical properties [31]. Meanwhile, the different mechanical properties of PANI/PLA-HA and PANI/PLA-SA may be related to the different density of the PANI doped by HCl and H_2_SO_4_, while the introduction of the APS in the reaction process can also have had a slight impact on the PLA nanofibers, with the comprehensive effect of these factors exhibiting different mechanical properties [32].

Cell adhesion, proliferation, and differentiation are affected by the morphology, with a rough surface generally believed to be conducive to cell adhesion [33]. The hydrophobicity of PLA nanofibers implies that the uniform polymerization of PANI presents a barrier, while the surface treatment of PLA nanofibers with plasma can significantly improve the wettability [34]. Following the PANI-based in situ polymerization with different inorganic acid dopants, PANI/PLA composite nanofibers with a uniform surface deposition were obtained.

**Fig. 3:**
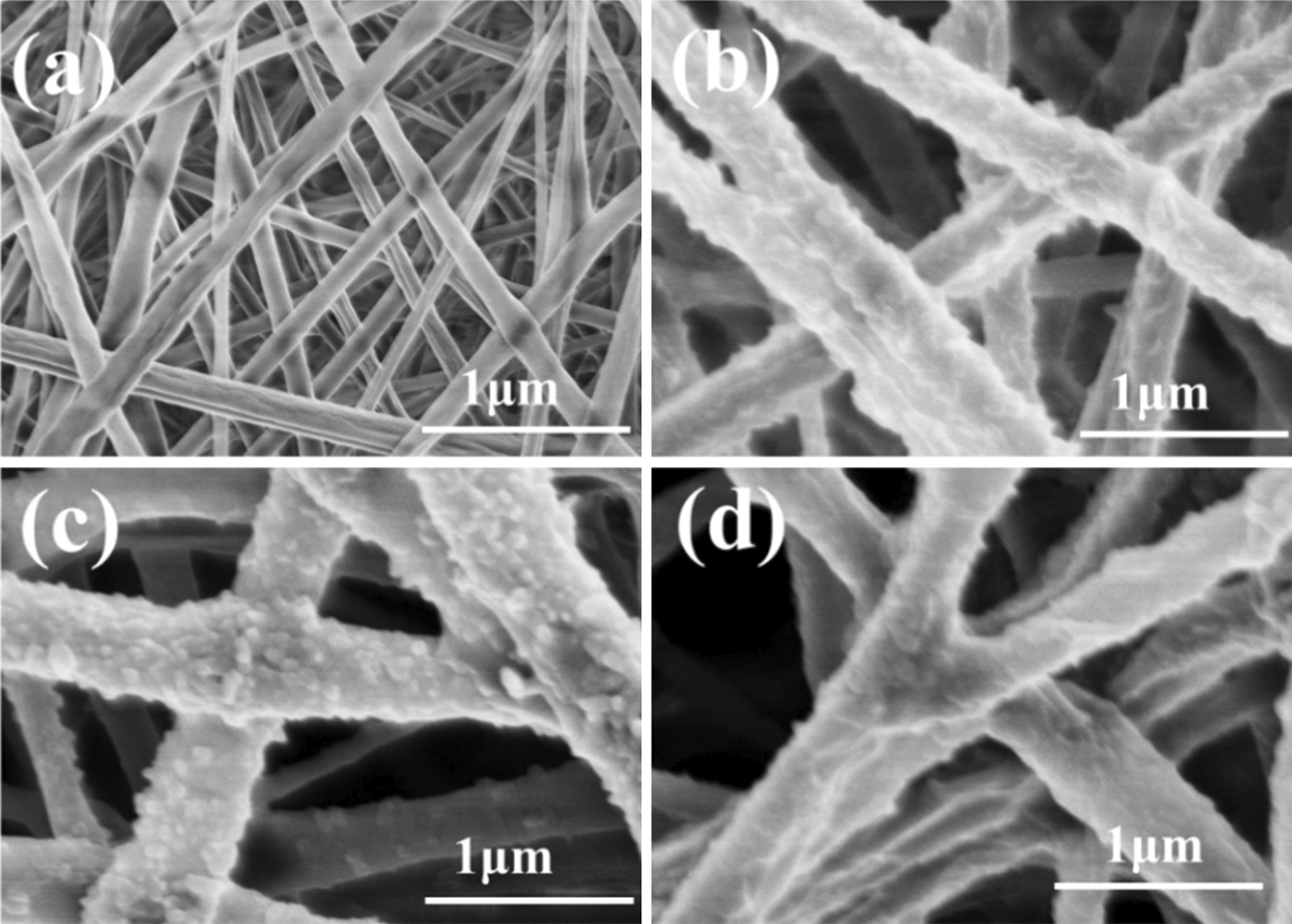
Morphology of **a** PLA nanofibers, **b** PANI/PLA-HA, **c** PANI/PLA-SA, and **d** PANI/PLA-PA composite nanofibers

**Fig. 4:**
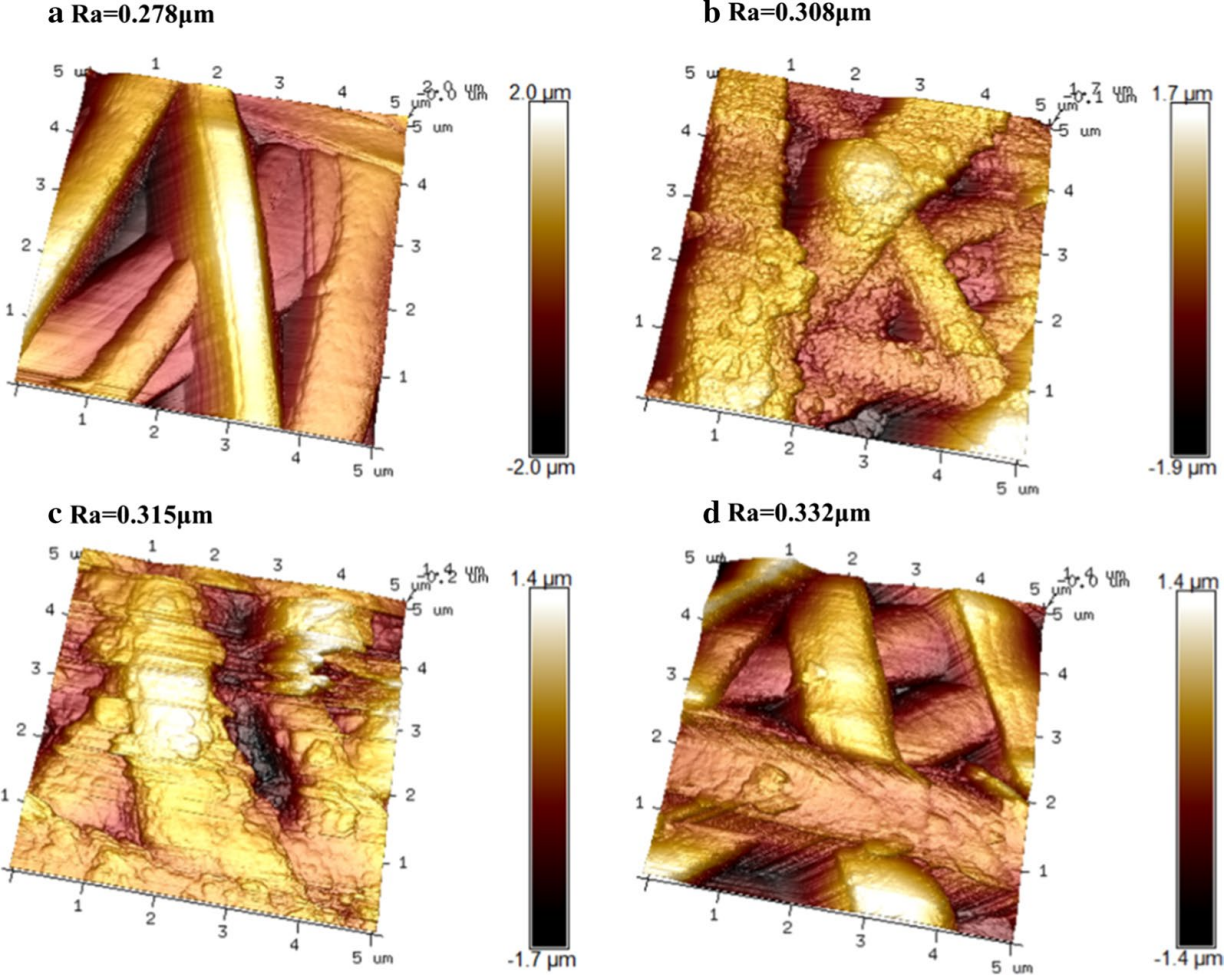
AFM image and surface roughness (Ra) of **a** PLA nanofibers, **b** PANI/PLA-HA, **c** PANI/PLA-SA, and **d** PANI/PLA-PA composite nanofibers

The PANI morphology on the surface of the different PANI/PLA fibers was observed via FE-SEM (Fig. 3). The figure clearly shows that the surface of the PLA nanofibers was covered by many irregular nanoparticles and that the PANI/PLA composite nanofibers doped with the inorganic acids were able to maintain good fiber morphology and porous nanofiber structure. The morphology observations revealed that the PANI/PLA composite nanofibers were successfully loaded with PANI, which provided a basis for cell adhesion and proliferation. Meanwhile, AFM was used to measure the surface roughness of the PANI/PLA composite nanofibers, as shown in Fig. 4. Ra, the mean value of surface roughness at three different positions of each sample, is generally used to evaluate sample’s surface roughness. Furthermore, the Ra of the PANI/PLA composite nanofibers was greater than that of the PLA nanofibers, and the Ra of PANI/PLA-PA was the highest. This increase in surface roughness accelerated the surface area and polarity, potentially providing more growth sites for cells and promoting cell adhesion.

**Figure. 5:**
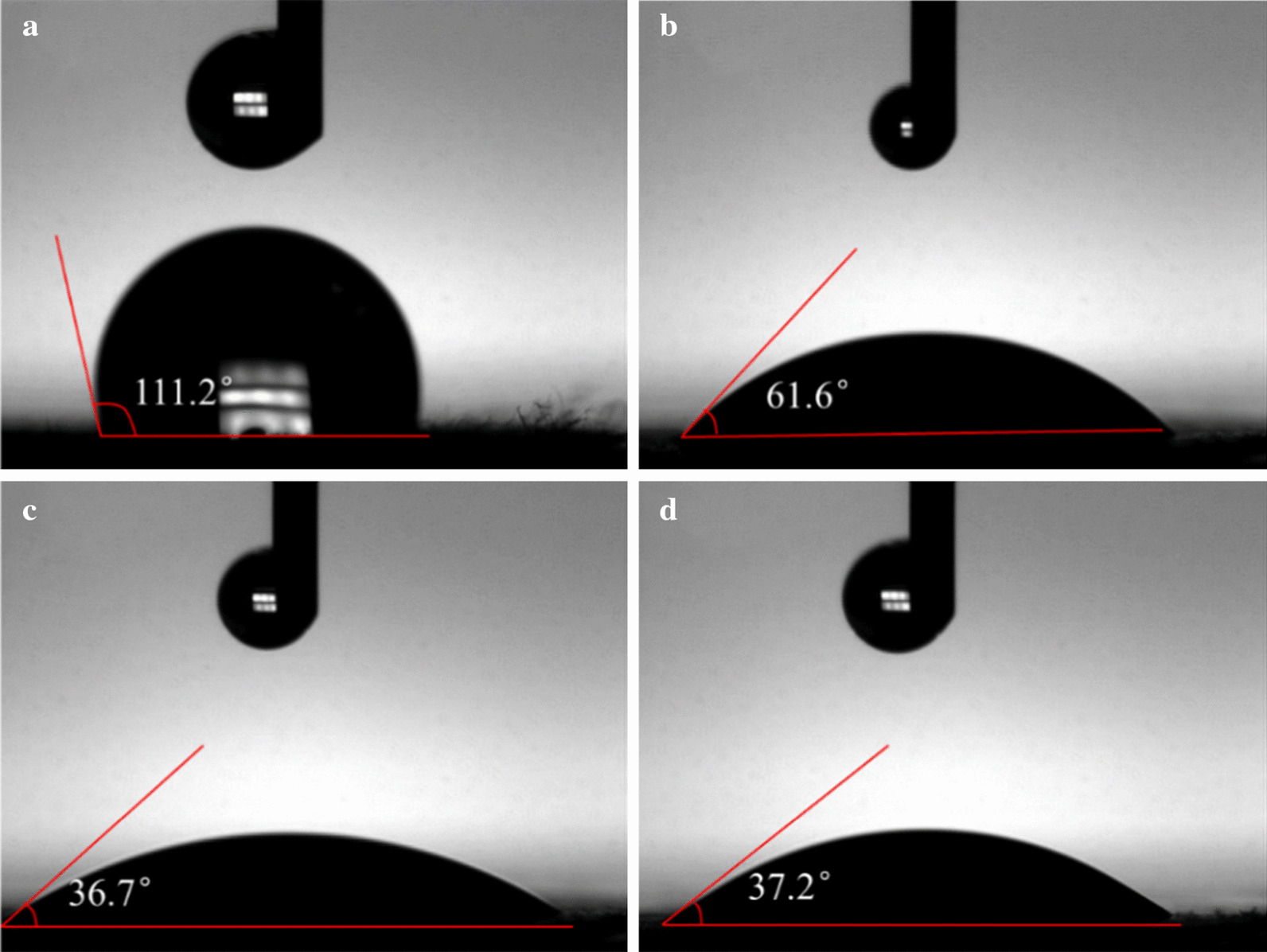
Contact angle of **a** PLA nanofibers, **b** PANI/PLA-HA, **c** PANI/PLA-SA, and **d** PANI/PLA-PA composite nanofibers

Cell adhesion, migration, and proliferation are significantly affected by the wettability of the scaffolds [35, 36]. In general, wettability is evaluated in terms of the contact angle between the scaffold and water. Given that PLA is hydrophobic, we measured the contact angle of the water droplets on the nanofiber membrane within 1 s, as shown in Fig. 5, and the contact angles of the PANI/PLA nanofibers following treatment were found to significantly decrease. The corresponding contact angles of PLA, PANI/PLA-HA, PANI/PLA-SA, and PANI/PLA-PA were 112°, 61.6°, 36.7°, and 37.2°, respectively. The PANI morphology of PANI/PLA increased the surface energy of the system, with the contact area increasing at the initial contact with the water, resulting in the decrease in the contact angle and the improvement of wettability. The contact angle of the composite nanofibers changed to 0° after 5 s of contact with water, thus demonstrating good hydrophilicity. This hydrophilic scaffold also provided favorable conditions for cell adhesion and diffusion [37] since the oxygen-containing functional groups (e.g., –OH and –COOH) on the PLA surface were more bound to the nanofiber surface following the plasma treatment, and the PANI morphology and oxygen-containing functional groups worked together to ensure the PANI/PLA composite nanofibers were ultimately completely wetted [38, 39].

**Fig. 6:**
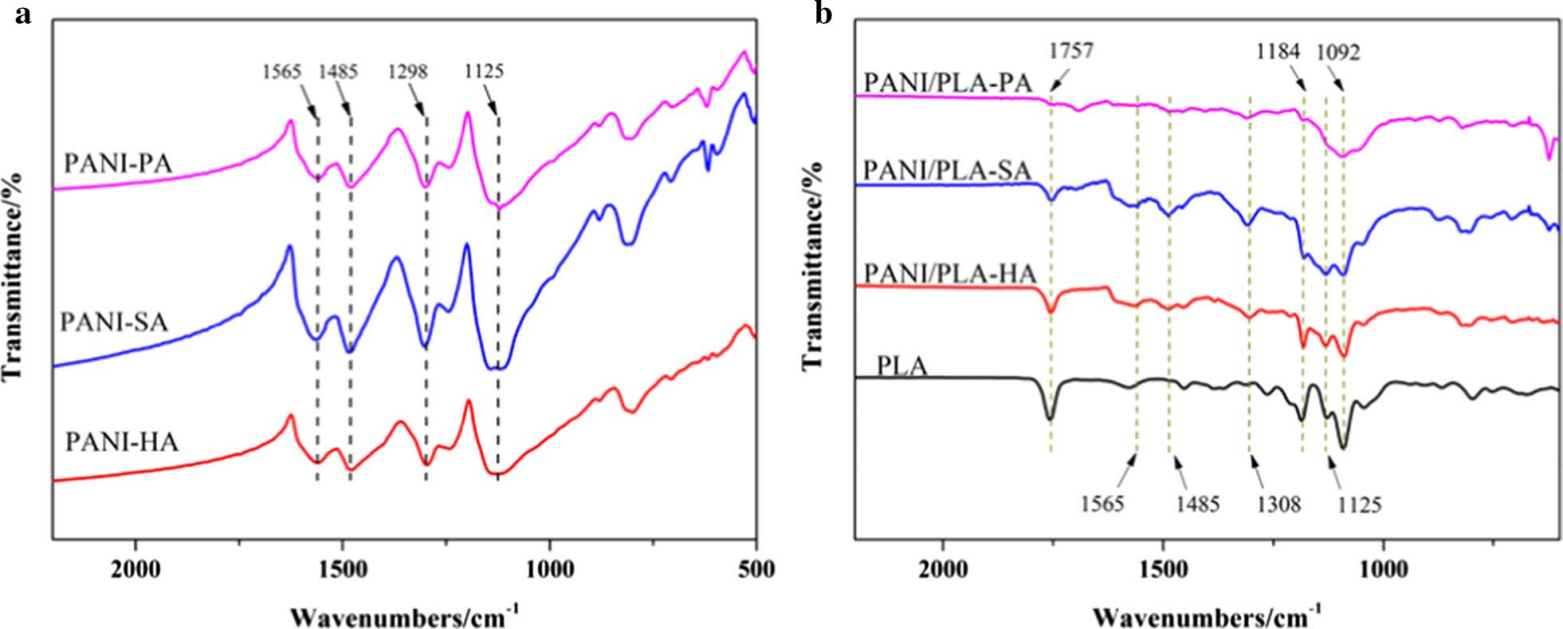
FTIR spectra of **a** PANI, **b** PLA and PANI/PLA composite nanofibers

**Fig. 7:**
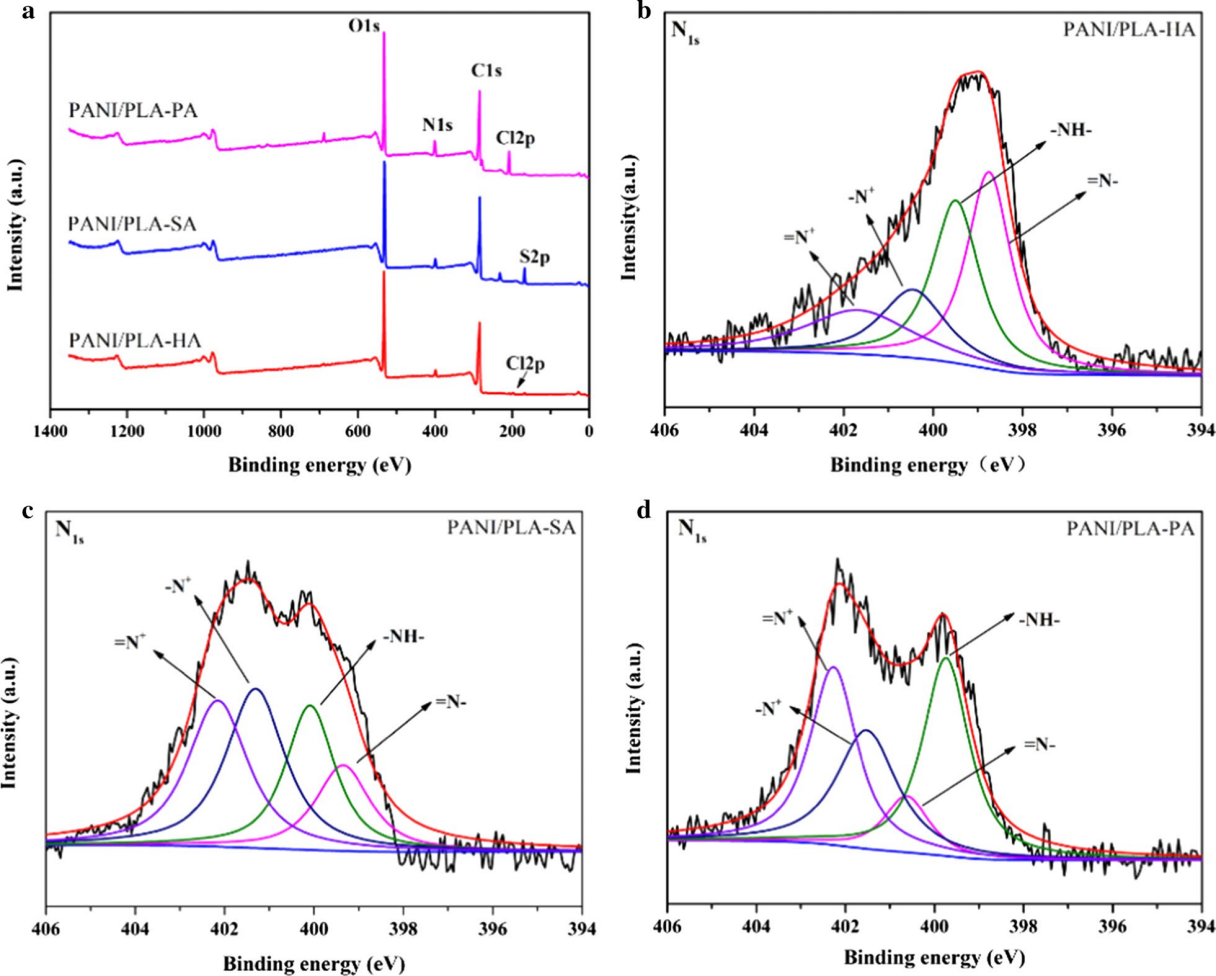
XPS spectra (**a**) of prepared PANI/PLA composite nanofibrous scaffolds and the PANI/PLA-HA (**b**), PANI/PLA-SA (**c**), and PANI/PLA-PA (**d**) of the core level signal of N1s

The FTIR spectra of pure PANI and PANI/PLA composite nanofibers doped by different inorganic acids are shown in Fig. 6. In the pure doped PANI spectrum (Fig. 6a), the strong characteristic peaks at 1,565, 1,485, 1,298, and 1,125 cm^−1^ correspond to a C=C stretching of the quinoid rings and a C = C stretching, C–N stretching, and = C–H stretching of the benzenoid rings, respectively. In the pure doped PANI spectrum (Fig. 6b), in addition to the characteristic PANI peak, a PLA peak can also be seen (C–O stretching vibration peaks of 1092 and 1184 cm^−1^, C=O stretching vibration peak of 1757 cm^−1^). These results indicate that PANI was successfully loaded on the surface of the PANI/PLA nanofibers doped with inorganic acids. To further investigate the chemical composition of the PANI/PLA nanofibers, XPS was used to analyze their surface composition. In addition, in the XPS spectra (Fig. 7a), clear N1s peaks were visible at ~ 400 eV in PANI/PLA composite nanofibers. Moreover, Cl2p peaks were visible at ~ 200 eV in PANI/PLA-HA and PANI/PLA-PA, while the peak intensity of Cl2p with PANI/PLA-PA was higher than that with PANI/PLA-HA. A peak of S2p appeared at ~ 210 eV on the XPS spectra in PANI/PLA-SA. XPS spectra indicated that Cl^−^, SO_4_^2−^, and ClO_4_^−^ were doped onto the corresponding PANI/PLA nanofibers. In addition, the imine nitrogen atoms of PANI were completely or partially oxidized to produce a series of oxidation states accompanied by varying degrees of protonation. The changes in the oxidation state and the protonation level of PANI were measured in terms of N1s nuclear level spectra (Fig. 7b–d). Each N1s spectrum can be deconvoluted to four major components with binding energies approximately 398.7, 399.6, 400.4, and 401.8 eV, which can be attributed to the quinonoid imine (–N=), benzenoid amine (–NH–),protonated amine (–N^+^), and protonated imine (=N^+^), respectively [40, 41]. With reference to Kumar’s study [42], the fitting peak of the N1s spectrum was deemed to be affected by the charge in the anions bound by the protonated N atoms, which resulted in delocalization and a slight shift.

**Fig. 8:**
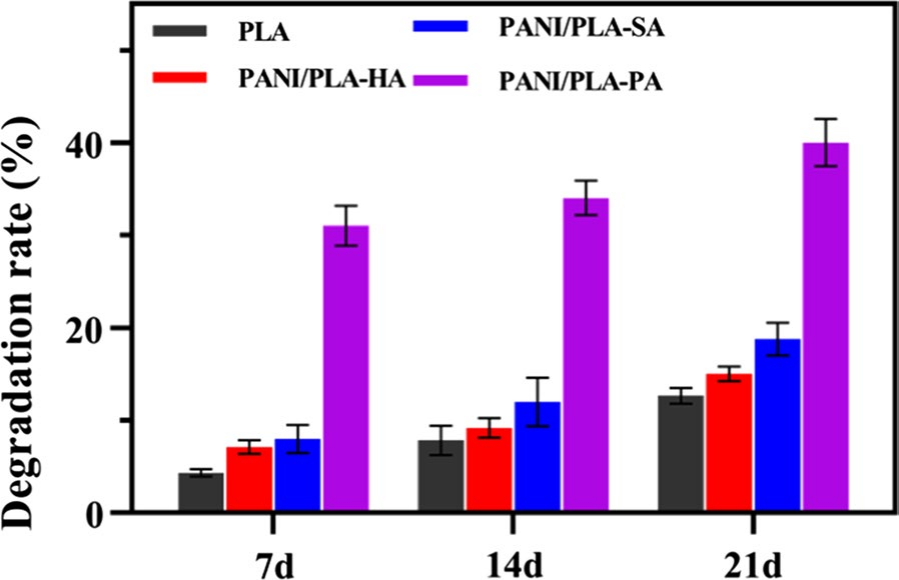
Degradation properties of PLA and PANI/PLA nanofibers

As a template for tissue repair and regeneration, bioactive scaffolds are degraded and excreted out of the body following induced cell and tissue repair [43]. In this paper, the degradation properties of the nanofiber scaffolds were evaluated using a mass loss method, as shown in Fig. 8. The mass loss of all samples increased at 7, 14, and 21 days, and the mass loss rates of the PLA nanofibers were 4.34 ± 0.41%, 7.84 ± 1.57%, and 12.65 ± 0.83%, respectively. Meanwhile, the mass loss of the PANI/PLA-PA composite nanofibers gradually increased following the in situ oxidative polymerization, with the mass loss rates of 31 ± 2.15%, 34 ± 1.86%, and 40 ± 2.54% at 7, 14, and 21 days, respectively, which were significantly higher than those of both PANI/PLA-HA and PANI/PLA-SA nanofibers. In the PANI in situ oxidative polymerization process, the presence of the oxidant APS could have destroyed the ester bond in PLA and induced a hydrolysis reaction, resulting in microcracks in the PLA nanofibers. With the prolongation of the PBS immersion time, the microcracks gradually accumulated and the PLA matrix began to gradually degrade. The surface-loaded PANI also fell off, resulting in a loss rate of nanofiber quality. With the increase in time, the mass loss ratio became clearer. Here, the strong oxidation of HClO_4_ aggravated the degradation of PLA and accelerated the mass loss of the PANI/PLA-PA nanofibers, which is consistent with the mechanical properties presented in Fig. 2.

The biocompatibility of bioactive scaffolds is the basis for promoting cell adhesion, growth, and proliferation [44]. Herein, we studied the cell proliferation of HOS on PLA and PANI/PLA composite nanofibers to illustrate the attendant adhesion and biocompatibility. During the chemical treatment and functionalization [45], a number of potential influencing factors could come into play in the preparation of bioactive scaffolds. Therefore, investigating their biocompatibility is key to evaluating their practical application.

**Fig.9:**
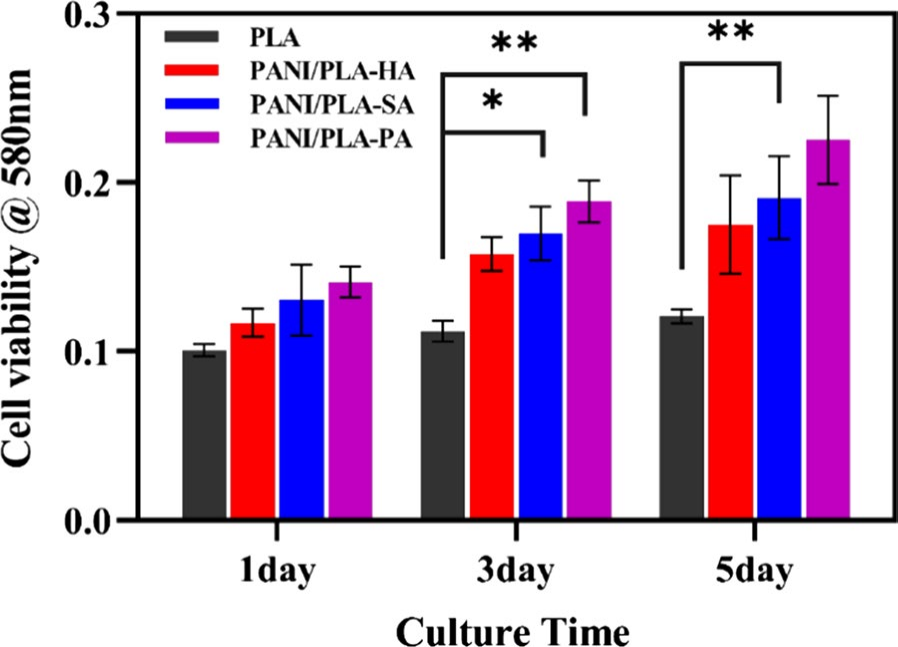
Cell viability HOS cultured for 1, 3, and 5 days on PLA nanofibers and PANI/PLA composite nanofibers (*p < 0.05; **p < 0.005)

To investigate the biocompatibility of PANI/PLA composite nanofibers, their cell viability was evaluated using a MTT method. Figure 9 shows the cell activity incubated on PLA and PANI/PLA composite nanofibers after 1, 3, and 5 days. The figure clearly shows that with the prolongation of the incubation time, the cell activity of the nanofibers gradually increased; the PANI/PLA-PA cells exhibited the best activity, and the cell activity following a five-day culture was the highest.

PLA is biodegradable, but hydrophobic, meaning that it is not conducive to cell adhesion, growth, and proliferation. Following the plasma treatment, the surface of the PANI/PLA composite nanofibers was loaded with oxygen-containing groups, and the functional surface demonstrated good hydrophilicity. The above morphology and AFM results indicate that the PANI doped with different inorganic acids exhibited different morphologies and roughness levels on the surface of the PLA nanofibers. Meanwhile, the PANI/PLA composite nanofibers exhibited excellent wettability. Therefore, we considered that the different morphologies of PANI doped with inorganic acids led to the enhancement of the surface energy and polarity of the PANI/PLA composite nanofibers, which consequently affected the cell growth, migration, and proliferation, resulting in improved performance in terms of cell activity [46].

**Fig. 10:**
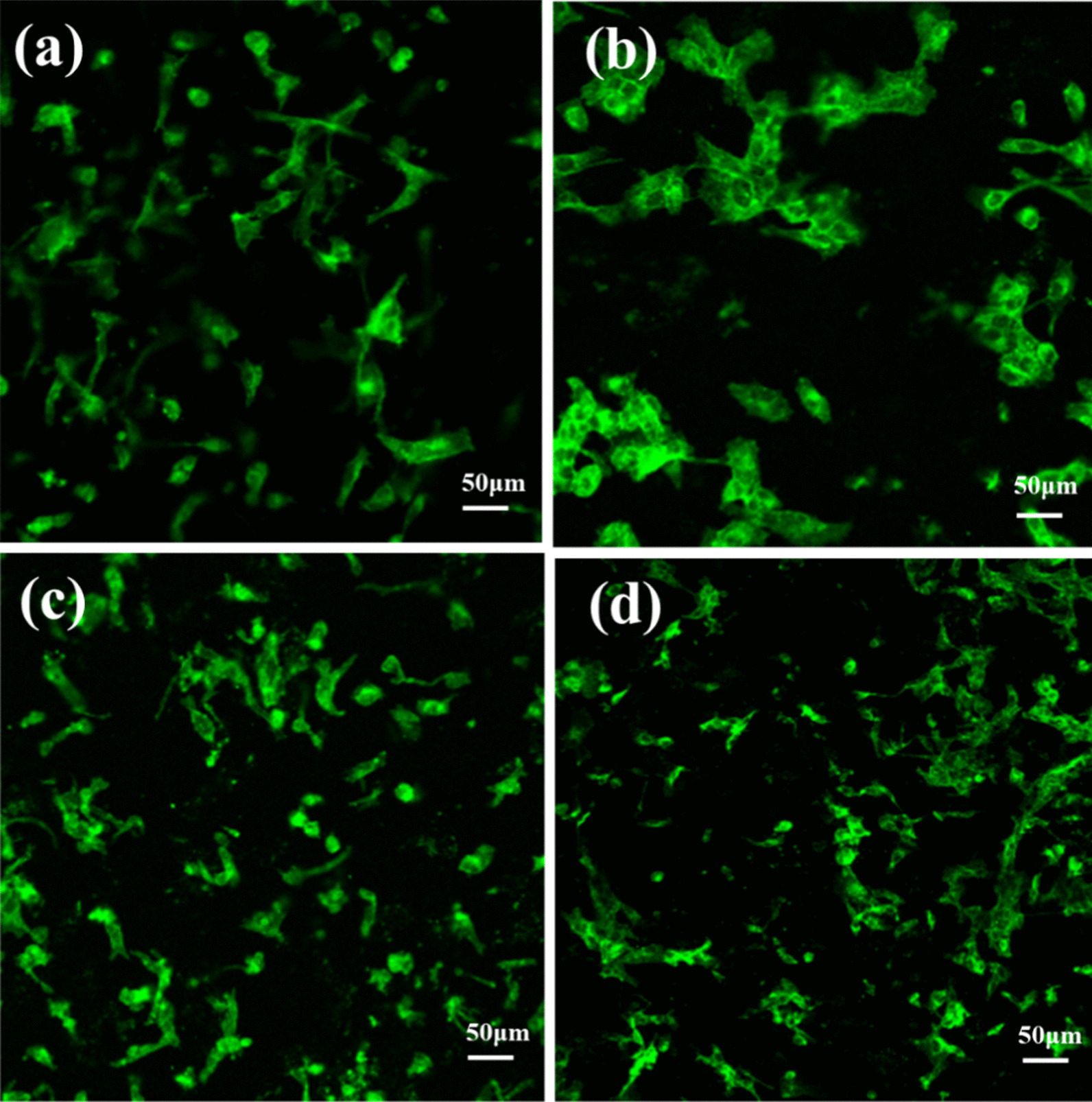
Fluorescence micrographic images on **a** PLA nanofibers, **b** PANI/PLA-HA, **c** PANI/PLA-SA, and **d** PANI/PLA-PA composite nanofibers after the incubation of 24 h

**Fig.11:**
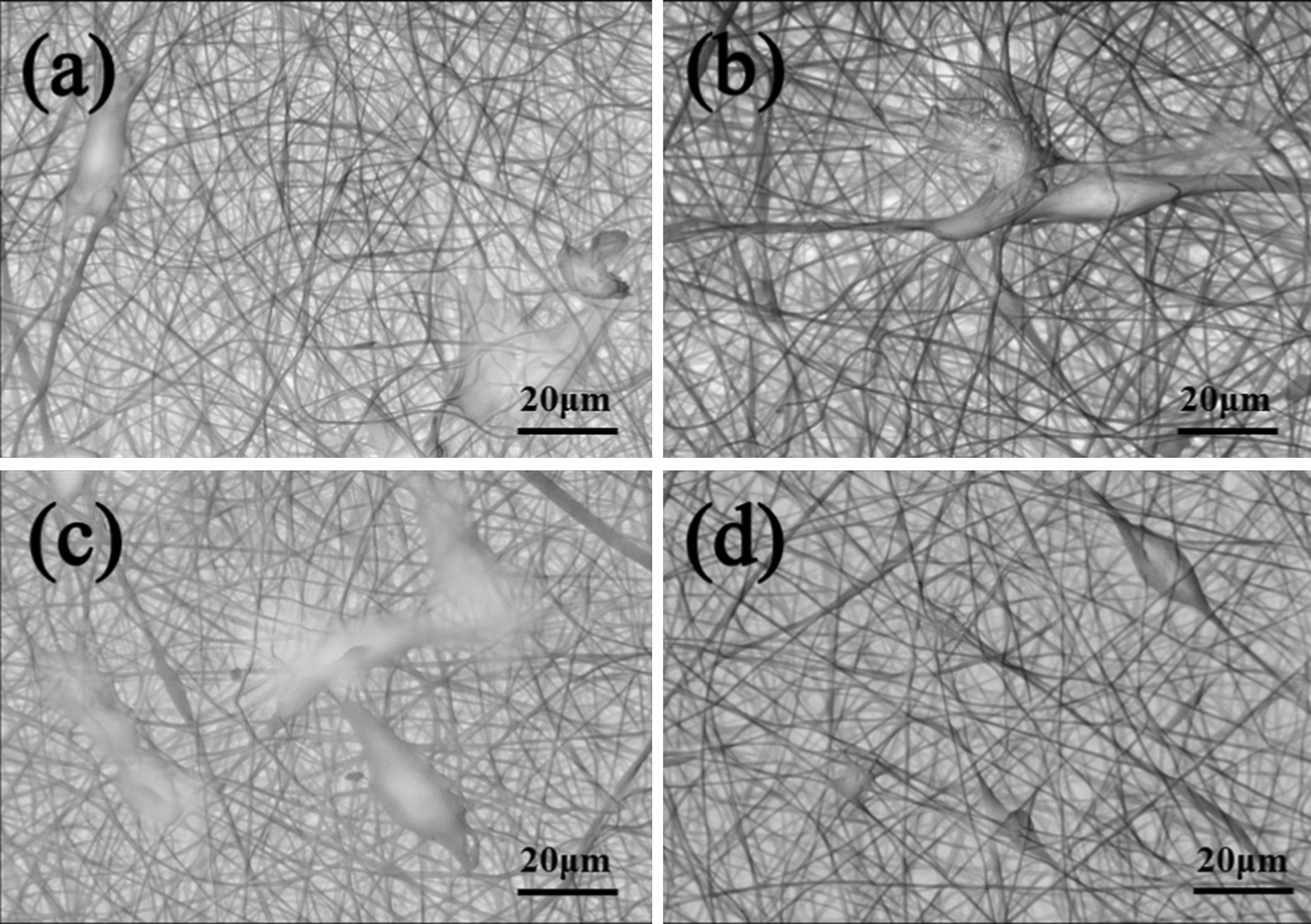
SEM micrographs of HOS seeded on **a** PLA nanofibers, **b** PANI/PLA-HA, **c** PANI/PLA-SA, and **d** PANI/PLA-PA composite nanofibers after 24 h

To further study the cell behavior of the PANI/PLA composite nanofibers, the growth and adhesion on the nanofibers were observed via fluorescence immunostaining (Fig. 10) and SEM (Fig. 11). Here, we compared the actin and cell morphology on the surface of different nanofibers. When the cells grew on the PLA fibers and PANI/PLA nanofibers, the actin bundles demonstrated a good stretching state. Meanwhile, cell density of the PANI/PLA composite nanofibers was higher than that of the PLA nanofibers of the control group, with the cell growth density following order PANI/PLA-PA > PANI/PLA-SA > PANI/PLA-HA. The HOS cells grew on the PANI/PLA nanofibers and adhered in a flat multipolar shape. Clearly, many cells were embedded in the pores of the PANI/PLA fibers but were poorly stretched on the PLA nanofibers and could not be fully expanded. These results indicate that PANI/PLA composite nanofibers could promote the adhesion and proliferation of HOS cells.

Meanwhile, the cell immunofluorescence staining and cell adhesion results indicated that the different PANI morphologies on the surface of the PANI/PLA composite nanofibers affected the growth, adhesion, and proliferation of the HOS cells, which was consistent with the above results.

**Fig. 12:**
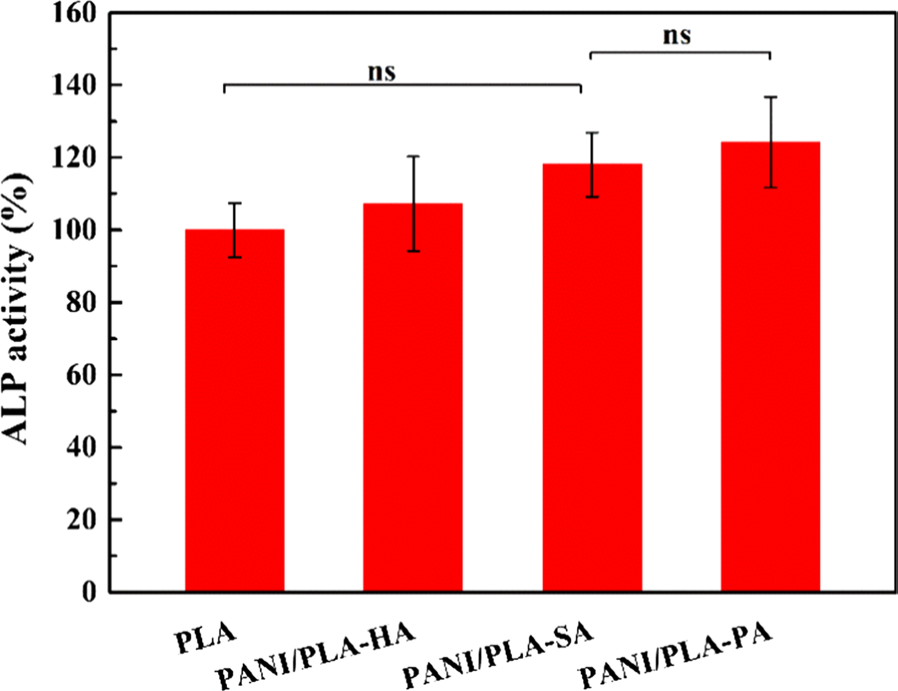
Alkaline phosphatase activity on PANI/PLA composite nanofibers scaffolds (ns = no significance)

As an early osteogenic marker, the ALP test was conducted on PLA and PANI/PLA composite nanofibers scaffolds for 7 days. Compared to the pure PLA nanofibers, the result (Fig. 12) showed that the ALP activity was significantly improved in of PANI/PLA composite nanofibers. Obviously, the ALP activity of PANI/PLA-PA composite nanofibers is the best. These results proved that PANI/PLA composite nanofibers exhibited better biocompatible, which is consistent with the above results of cell adhesion, growth, and proliferation.

## Conclusions

In this paper, PANI/PLA composite nanofibers of different surface morphologies were prepared by three types of inorganic acid as dopant in in situ polymerization. We confirmed that PANI could be successfully loaded on the surface of PLA without changing the porous structure of the nanofibers. The mechanical properties and in vitro degradation experiments demonstrated that oxidizing acids can significantly weaken the mechanical properties and accelerate the degradation of polyester nanofibers. Meanwhile, the rougher surface resulted in a better wettability and promoted the cells adhesion, growth, and proliferation, which indicated a better biocompatibility. In conclusion, the regulated PANI morphology via different acids doping has positive effect on biocompatibility in tissue engineering.

## Data Availability

The authors declare that the materials and data are promptly available to readers without undue qualifications for material transfer agreements. All data generated or analyzed during this study are included in this article.

## Abbreviations

PANI: Polyaniline
PLA: Polylactic acid
ECM: Extracellular matrix
PPy: Polypyrrole
PTH: Polythiophene
AN: Aniline
DCM: Dichloromethane
DMF: *N*,*N*-Dimethylformamide
APS: Ammonium persulfate
HOS: Human osteosarcoma cells
DMEM: Dulbecco’s modified Eagle medium
PBS: Phosphate buffer saline
MTT: 3–2,5-Diphenyl-2-H-tetrazolium bromide
FITC: Fluorescein isothiocyanate
FTIR: Fourier transform infrared spectroscopy
FE-SEM: Field emission scanning electron microscopy
XPS: X-ray photoelectron spectroscopy
ALP: Alkaline phosphatase
